# Self-evaluation of present clinical skills by medical students in the years 3 to 6 – a pilot study in four European countries

**DOI:** 10.3205/zma001182

**Published:** 2018-08-15

**Authors:** Leonard Westermann, Barbara Zisimidou, Marvin Simons, Rene Zellweger, Dominik Baschera

**Affiliations:** 1 University Hospital Cologne, Department for Orthopaedics and Traumatology, Cologne, Germany; 2 Royal Perth Hospital, Department of Orthopaedics and Trauma Surgery, Perth, Australia; 3 Kantonsspital Winterthur, Department for Neurosurgery, Winterthur, Switzerland

**Keywords:** Basic medical skills, preparation for clinical practice, satisfaction of medical students

## Abstract

**Background: **Clinical training concepts of medical students differ in the various European countries. The goal of this paper is to study the differences at the beginning of medical practice in specific clinical skills on an international level.

**Methods:** The data were collected by a publically accessible online questionnaire online from February to June 2010. The participants in the study were recruited through the official letter sent by deaneries and student organisations. Two thousand nine hundred and seven medical students participated in the online survey. From study years 1 to 6, 2406 valid data records (67.3 percent female; 32.7 percent male) from four different European countries were sent. The skills in the questionnaire included patient consultation and anamnesis, physical examination, auscultation, gypsum and bandage dressing, suture techniques, venepuncture, and laying of indwelling catheters.

**Results: **One thousand six hundred and twenty-nine data records of medical students in their training years 3 to 6 were assessed. The average age of the students was 24.7 years. On a scale from 1 to 10, the average satisfaction of the students with their medical faculty was 6.47 (±2.07); the assessment of the preparation for the clinical activities was 4.72 (±2.13). By comparison, British students indicated most satisfaction with their training (6.70±1.85). With respect to the clinical skills, the students interviewed felt safest in patient consultation and anamnesis (7.63±2.13) followed by blood sampling (7.46±2.29). The topics of surgical suturing techniques (4.40±2.81) and the gypsum and bandaging techniques (2.63±2.23) were taught worst subjectively.

**Discussion: **The training of medical students in basic clinical skills is an essential part of the studies. This study was able to demonstrate that the subjective trust of medical students in their personal skills positively correlated with the satisfaction with their own university. The results pointed out that future curricula of universities could profit from an increased focus on clinical skills.

## Zusammenfassung

**Hintergrund: **Klinische Ausbildungskonzepte von Medizinstudenten unterscheiden sich in den verschiedenen europäischen Ländern. Ziel dieser Studie war es Unterschiede am Beginn der ärztlichen Tätigkeit in spezifischen klinischen Fähigkeiten im internationalen Vergleich zu untersuchen.

**Methodik: **Die Daten wurden im Rahmen eines öffentlich zugänglichen Onlinefragebogens im Zeitraum von Februar bis Juni 2010 erhoben. Die Rekrutierung der Studienteilnehmer erfolgte über das offizielle Anschreiben von Dekanaten und Studentenvereinigungen. 2907 Medizinstudenten nahmen an der Onlinebefragung teil. Aus den Studienjahren 1-6 stammten 2406 gültige Datensätze (67,3% weiblich; 32,7% männlich) aus vier verschiedenen europäischen Ländern. Bei den erfragten Fähigkeiten handelte es sich um Patientengespräche und Anamneseerhebung, körperliche Untersuchung, Auskultation, Gips- und Verbandsversorgung, Wundnahttechniken, Venenpunktion und das Legen von Venenverweilzugängen.

**Ergebnisse: **1629 Datensätze von Medizinstudenten in den Ausbildungsjahren 3-6 wurden ausgewertet. Das durchschnittliche Alter der Studenten betrug 24,7 Jahre.

Die durchschnittliche Zufriedenheit der Studenten mit ihrer medizinischen Fakultät lag auf einer Skala von 1 bis 10 bei 6,47 (±2.07), die Bewertung der Vorbereitung auf die klinische Tätigkeit betrug 4.72 (±2.13). Im Vergleich zeigten britische Studenten die größte Zufriedenheit mit ihrer Ausbildung (6.70±1.85). Von den klinischen Fähigkeiten fühlten sich die befragten Studenten am sichersten bei der Patientengesprächsführung und Anamneseerhebung (7.63±2.13), gefolgt von Blutabnahmen (7.46±2.29). Inhalte der chirurgischen Nahttechniken (4.40±2.81) und Gips- und Verbandstechniken (2.63±2.23) wurden subjektiv in allen Ländern am schlechtesten vermittelt.

**Diskussion: **Die Ausbildung von Medizinstudenten in klinischen Basisfähigkeiten ist ein essenzieller Bestandteil des Studiums. Diese Studie konnte zeigen, dass das subjektive Vertrauen der Medizinstudenten in die persönlichen Fähigkeiten positiv mit der Zufriedenheit in die eigene Universität korreliert. Die Ergebnisse weisen darauf hin, dass zukünftige Lehrpläne der Universitäten von einem vermehrten Fokus auf klinische Fähigkeiten profitieren könnten.

## Introduction

*“Knowing is not enough; we must apply. Willing is not enough; we must do.”* (Goethe)

Practice and mastery of basic clinical skills are fundamental prerequisites for the beginning of the medical profession. Many assistant doctors and medical trainers are in agreement that the personal repertoire of clinical skills also influences medical decisions [1].

Although many universities continue to pursue the Flexner model and focus on basic theoretical knowledge during the first two years, the so-called preclinical studies are nonetheless a period to provide the medical students with an introduction and a basis of clinical skills [1].

During the last few years, an increasing importance has been attached to early knowledge in basic medical skills in university education, in particular in the organisation of clinical traineeships and internships [2], [3]. Study results have revealed that many students do not feel sufficiently trained for practical clinical tasks during their clinical traineeship and for the beginning of their career. Various studies report that students have difficulties putting their theoretical knowledge into clinical practice and recognise deficiencies in the required basic skills in medical activities [4]. A study was able to demonstrate that a quarter of the medical students reported having difficulties transferring pre-clinical basic knowledge into clinical work [5].

The pre-clinical study section is intended to prepare the students to the clinical aspects of education. Radcliffe and Lester interviews of medical students during their final year of training reveal that this is characterised decisively by stress during the transitional period to the activities to medical activities. The students describe a feeling of “uselessness” and inability in medical treatment due to lacking knowledge at the beginning and insufficient clinical skills [6].

The fact that the implementation of clinical skills in the curricula of the preclinical phase correlates with an improved performance of students during clinical traineeships could be demonstrated in studies on students during their third year, in particular in the field of internal medicine [7]. Comparable curricula with a focus on clinical skills can lead to an improvements of the performance [7]. An increasing number of universities support patient contact during the first weeks of studies already [8]. The entire medical education, in particular learning medical basic skills, should be focussed on patients [9]. When training basic skills, the clinical context should be taken into consideration at an early stage already in order to understand the basic principles and the nature of the techniques [9].

The aim of the pilot study was to examine the obvious differences between the subjectively assessed basic skills of students in the participating European countries. The competencies assessed included patient consultation and anamnesis, physical examination, auscultation, gypsum and bandage dressing, suture techniques, venepuncture, and laying of indwelling catheters. 

The global satisfaction of the medical students with their own faculty and training in the assessed items was to be determined. In accordance with the previous literature, our hypothesis was that students feel unprepared for clinical everyday work due to lacking training of basic practical skill within the framework of their training.

## Methods

The data were acquired online using the freely accessible online programme Limesurvey (version 1.85 RC3) (see Attachment 1). The following data were included in this survey:

- The town and country of the medical university
- The age of the students
- The medical training year
- The sex
- The self-assessed satisfaction with the medical university (scale 1 to 10)
- The self-assessed state of preparation for clinical activities (scale 1 to 10)
- The self-assessed state of preparation for the requested seven clinical skills which are required during the practical year and at the beginning of the clinical everyday activities of doctors (patient consultation and anamnesis, physical examination, auscultation, gypsum and bandage dressing, suture techniques, venepuncture and laying of indwelling catheters) (scale 1 to 10).

The questionnaire was validated in several meetings with test runs involving medical students from various countries and various mother tongues. A formal pilot study ahead of this study was not carried out. In February 2010, the public questionnaire was published online in German and English. Academic deanships in Germany, Switzerland, Great Britain and Austria were contacted with the request to forward a link to their medical students. We received replies from students in their 1 to 6 years of training and a negligible number of replies from students in their bridge years.

In order to guarantee an improved comparison, we only analysed the clinical skills of medical students in their years 3 to 6. Reference points for the application of practical skills were clinical traineeships and the practical year in clinical distance to medical training.

The data were assessed by means of the SPSS21 statistics software. The statistics were taken separately comparing country, sex and study year.

The following non-parameterised tests were applied:

1. Kruskal-Wallis test used to compare the skills between the countries
2. Mann-Whitney U test used to compare the distribution according to sex.

The Pearson Product Moment and the Spearman’s Rank Order correlation was used to analyse the correlations between two continuous variables. The significance level was p<0.05.

## Results

All-in-all we received data records from 2,907 medical students between February and June 2010.

**Figure 1:**
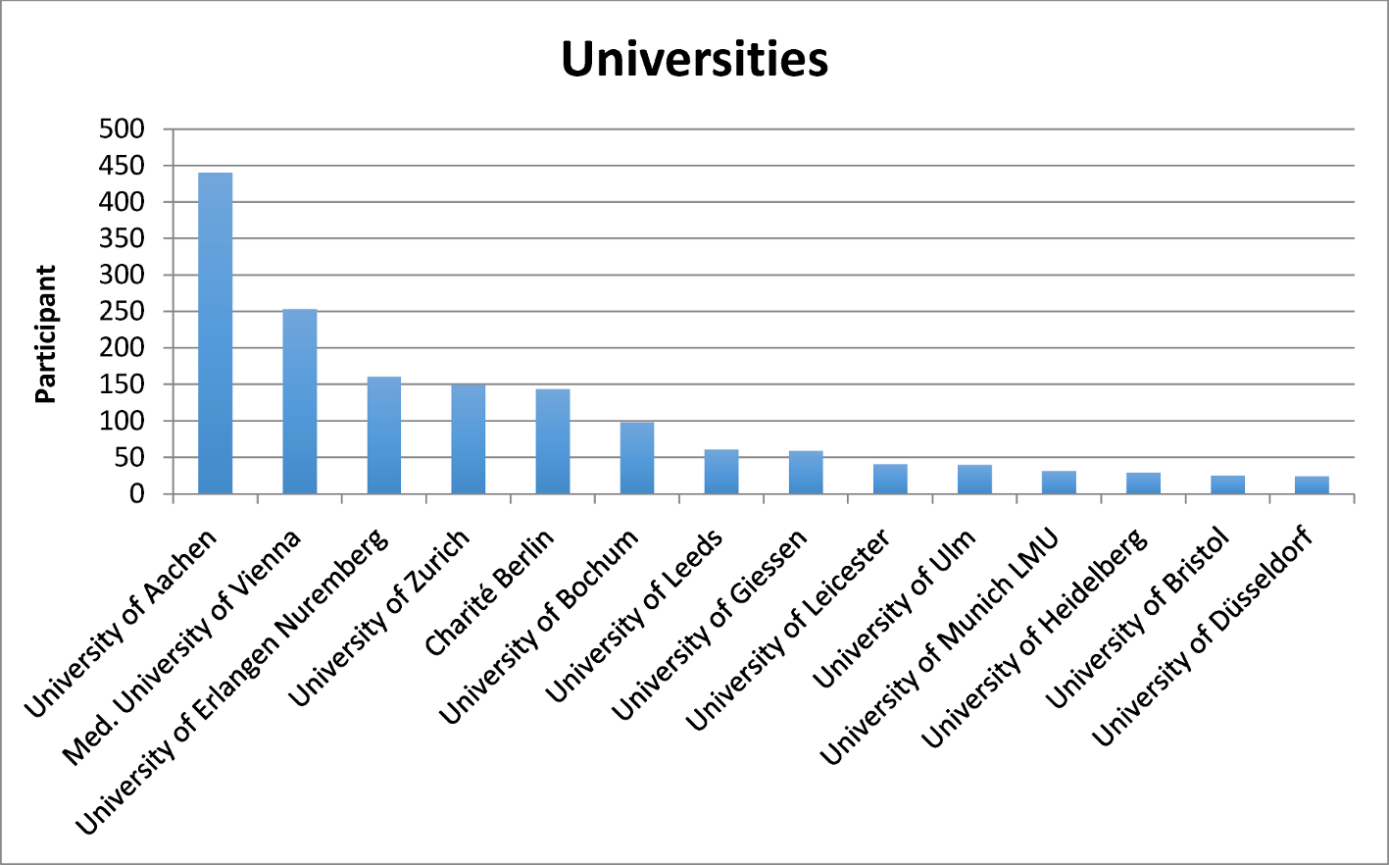
Participating medical faculties with the highest number of participants

Replies were received from 36 of the 44 German-speaking medical universities in Germany, Switzerland and Austria as well as from three medical faculties in Great Britain. After deducting incomplete data records without country code (n=501), we received in total 2,406 complete replies (1,662 from Germany (GER), 310 from Austria (A), 234 from Switzerland (CH), and 200 replies from Great Britain (UK). One thousand six hundred and twenty-nine data records were from medical students in their training years 3 to 6 (clinical years) (see Figure 1 (Fig. 1)).

Amongst the participating students there were 1,097 (67.3 percent) female medical students and 532 (32.7 percent) male medical students. The average age was 24.7 years (±3.1).

On a scale from 1 (dissatisfied) to 10 (very satisfied) the satisfaction of the students with their own medical faculty was 6.47 (±2.07) on average. On a scale from 1 (not especially good) to 10 (excellent), the preparation for practical work was assessed 4.72 (±2.13) on average (see Table 1 (Tab. 1)).

By comparison, the participating students from Great Britain stated better preparation for their clinical activities on average with 6.70 (±1.85) (1=not satisfied – 10 very satisfied) than students from Austria 5.29 (±2.24), students from Switzerland 4.70 (±1.88) and students from Germany who on average only stated 4.32 (±2.00) The results of the individual countries were significant.

The participating students from all countries indicated no significant differences with respect to the clinical skills of anamnesis and patient consultation, blood sampling and laying winged infusion sets (see Table 2 (Tab. 2)). The greatest uncertainties were stated in surgical suturing techniques and gypsum and bandaging techniques.

A positive correlation was revealed between blood sampling and laying winged infusion sets (0.798) auscultation and the physical examination (0.728) as well as physical examination and patient consultation (0.544).

**Figure 2:**
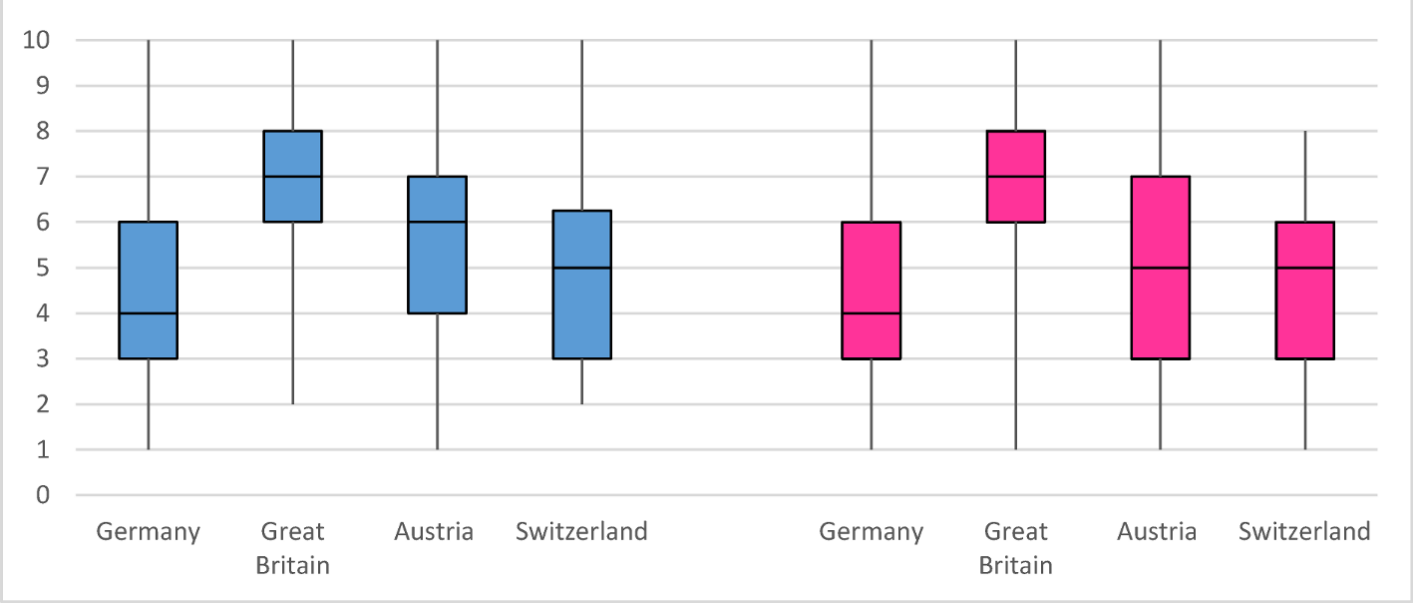
Assessment of the preparation by the medical faculty for clinical activity comparing male students (left) and female students (right) on a scale from 1=very bad to 10=very good

Figure 2 (Fig. 2) displays the comparison between self-assessments of female and male medical students in the clinical skills queried. Male students replied with 4.99±2.11 and female students with 4.72±2.19 (p=0.008) to the question “How well do you feel prepared for the clinical activity by your medical faculty ?” (see Table 3 (Tab. 3)).

It appears that a positive self-assessment of own skills also correlated to a better assessment of own studies (cf. Table 4 (Tab. 4)).

Lower to moderate correlations were found between the assessment of the training in the skills and the self-assessed performance. 

## Discussion

This pilot study analysed the self-assessment of medical students in their third to sixth training years with respect to mastery of seven predefined basic skills. It is generally clear that the students in all participating countries feel most secure during patient consultation and when performing structured physical examination. Most students expressed uncertainty in clinical skills, such as performing a surgical suture or applying gypsum/bandage. This study could prove a moderate satisfaction of the medical students with their medical faculties.

By comparison, medical students from Great Britain revealed a higher self-assessment with respect to their clinical preparation. As already mentioned in the present literature, a curriculum which contains early clinical training already will lead to improved performance and self-confidence in internships, clinical traineeships and when starting the clinical profession [7], [8], [10]. This study reveals that a large number of the participating student seem to feel insecure when carrying our essential clinical skills. Slight differences could be found in international comparison with respect to preparation. Some factors could have had an influence on the results of satisfaction with the clinical training of medical students from Great Britain. Many medical faculties in Great Britain and, in the meantime, all over the world, have adopted the recommendations of the General Medical Council (GMC) which recommends an early confrontation with clinical abilities and thus reports about an improved outcome [11], [12].

Howard Barrows, who had developed the well-known principles of Problem-Based Learning (PBL) in the 1960s, suggests that students learn more confidently and directly with a problem-oriented curriculum [13]. PBL improves the use of basic theoretical knowledge for clinical questions [14]. This model wide-spread in Great Britain may explain the improved self-assessment with respect to the clinical training of medical students.

Our data seem to underline the results of Katinka et al. and Whipple which reveal that students feel confident in anamnesis and physical examination [4], [10] (physical examination and auscultation were assessed as safest skills).

The Association of American Medical Colleges (AAMC) recommends that supervised patient interaction should take place frequently at an early stage in order to improve the preparation for patient-oriented care in everyday clinical work [9].

The review of curricula and the corresponding reforms are thus sensible. In view of the integration of early clinical skills, an improved co-operation between the individual faculties and the responsible persons in teaching is indispensable [15].

The authors are of the opinion that advanced clinical skills, such as surgical suture techniques and gypsum techniques, should be included in clinical training at an early stage already. Although these are specific techniques of some disciplines, but the principles of these should be taught as well. On account of an improved communication between the preclinical and clinical subjects with respect to clinical practice, medical students would profit [12], [15].

Nonetheless, continuing efforts are required to improve clinical training for practice and to evaluate the best methods of the various clinical “skills programmes” [7].

With respect to the results it has to be noted that they are subject to the usual limitations of the method chosen. The difference of the number of participants from the various countries caused by the study design complicates an exact international comparison.

The interpretation of the results lets us assume that the assessment of the training of the medical students for subsequent clinical activities and the lower satisfaction with the medical faculty correlates the security in the performance of clinical skills examined. The results suggests that medical curricula in future could profit from an increased focus on clinical skills which apart theoretical knowledge are a basis for everyday clinical activities at a later point. The improved self-confidence of the medical students in everyday clinical activities would lead to a better ranking in the satisfaction with the medical faculty. 

Similar studies with a higher number of cases, isolated to the German-speaking area, would permit a comparison of the different curricula and their influence on the clinical training.

## Key message

1. In an international comparison, important aspects for anamnesis and patient consultation are taught well during the studies already. These basic skills are subjectively mastered safely at the beginning of the medical activities.
2. Basic surgical skills, such as suturing techniques and gypsum and bandaging technique are taught worst during studies even in an international comparison.
3. Male students mastered all basic practical skills safer than their female fellow students. In anamnesis and patient consultation, female students demonstrated more self-confidence than their male students.
4. It remains to be assumed that an increased self-confidence leads to a better assessment of the medical faculty.

## Competing interests

The authors declare that they have no competing interests. 

## Supplementary Material

Survey

## Figures and Tables

**Table 1:**
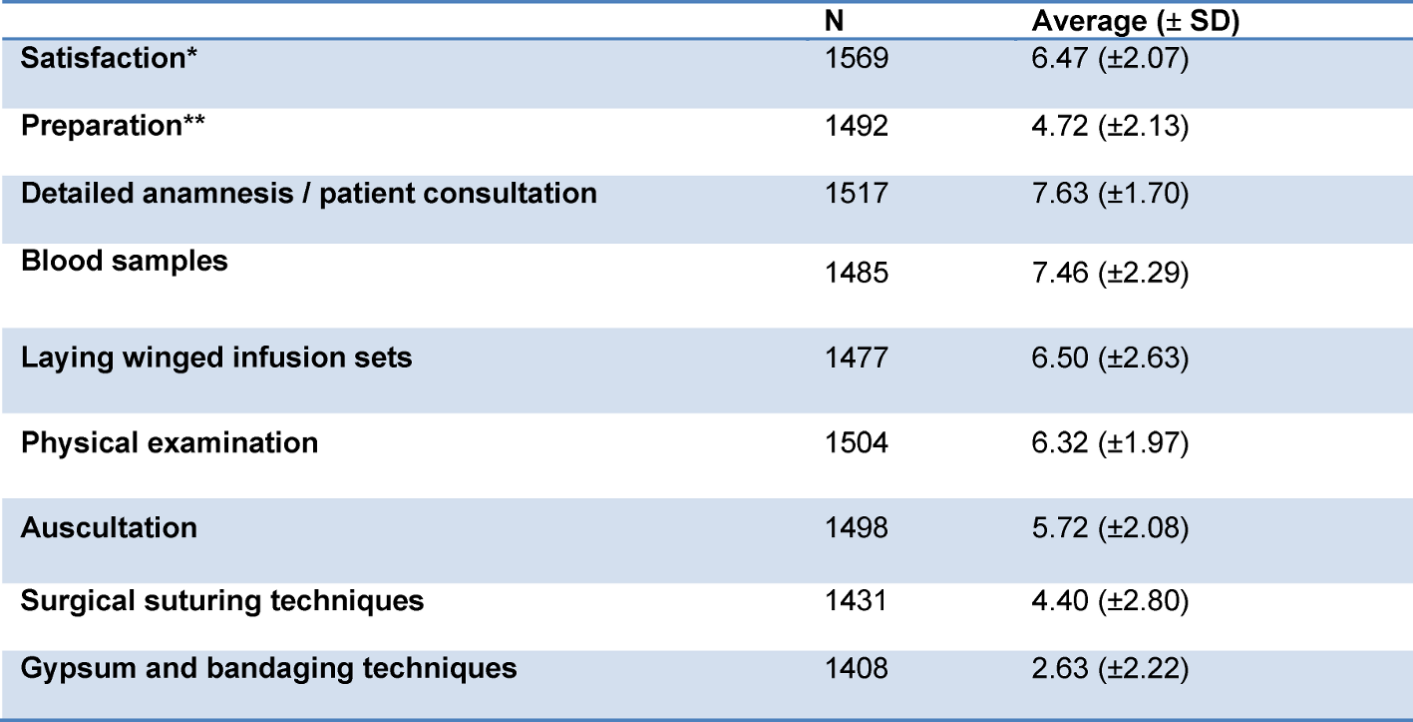
Summary results for all countries participating. *Satisfaction with own medical faculty, **Preparation by own medical faculty for the beginning of clinical work. Average on a scale from 1=very bad to 10=very good), N: Number, SD: Standard deviation

**Table 2:**
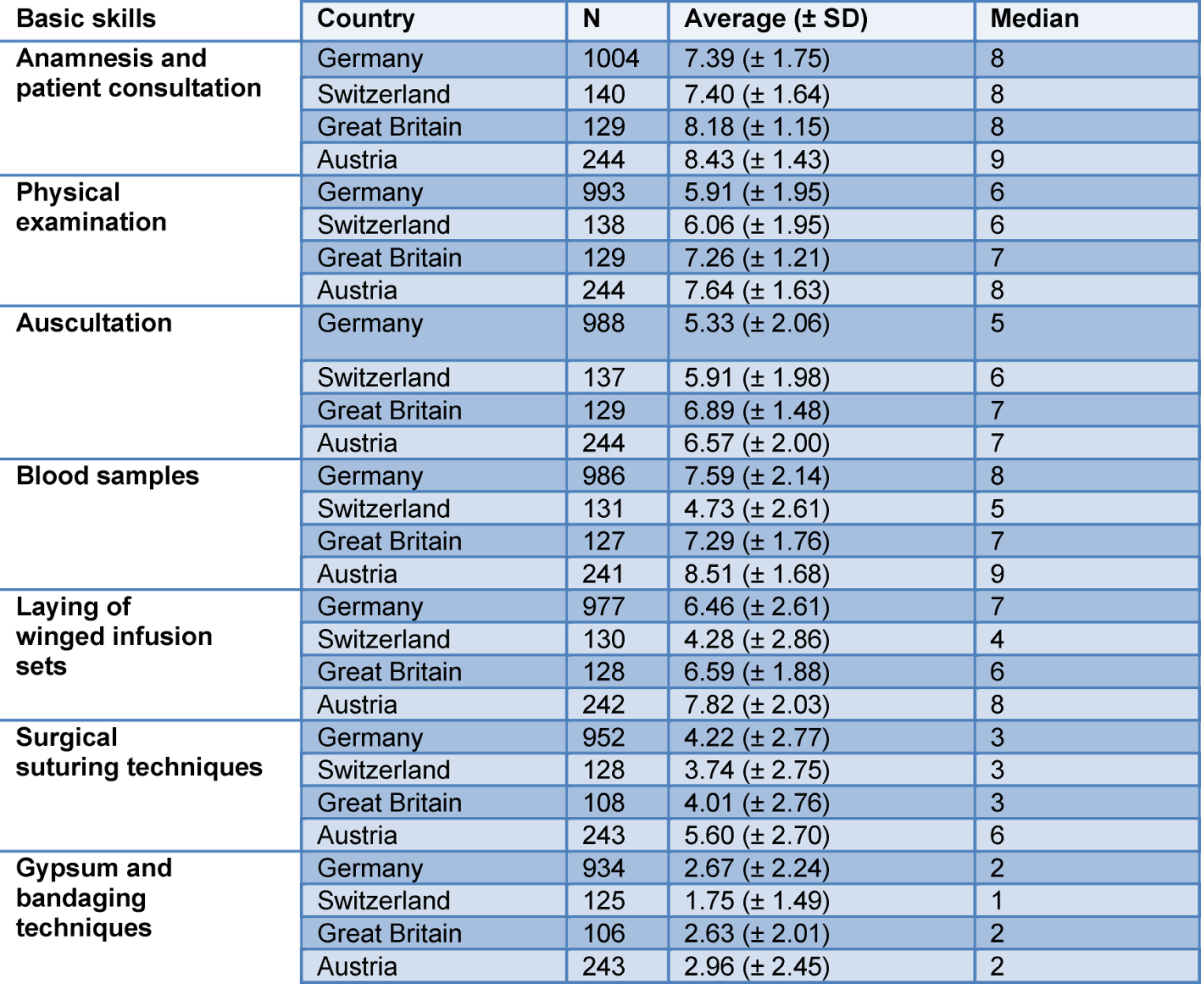
Confidence in clinical skills according to countries. Average on a scale from 1=very bad to 10=very good), N: Number, SD: Standard deviation

**Table 3:**
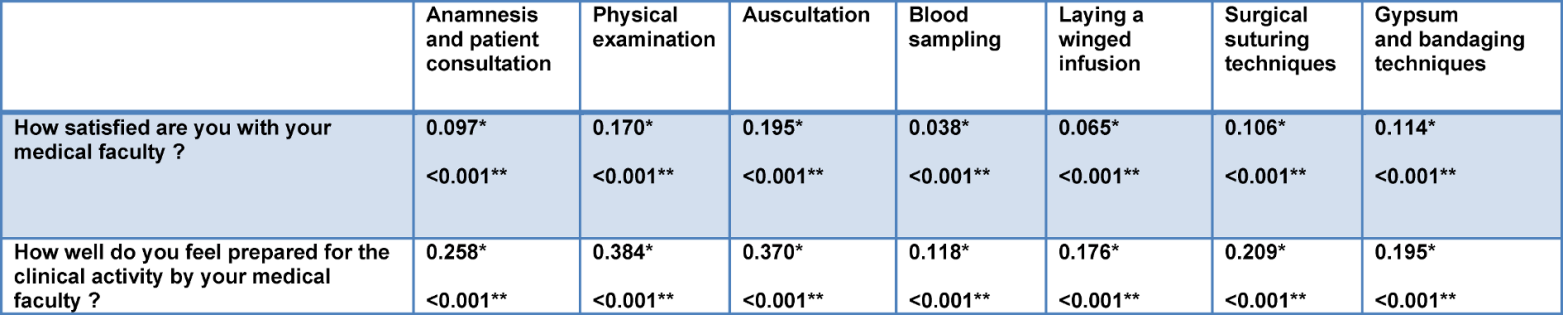
Spearman’s rho correlation between satisfaction with the faculty/preparation for the clinical skills. * Spearman’s rho correlation coefficient, **Significance

**Table 4:**
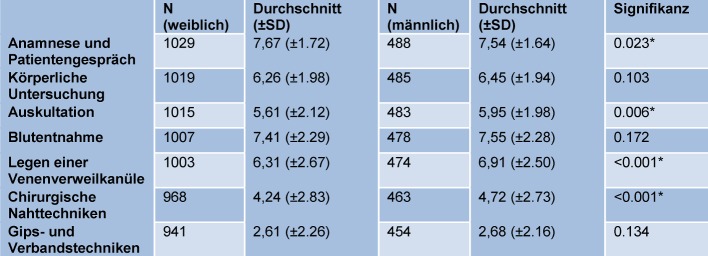
Confidence in clinical skills and gender-specific disparities. Average on a scale from 1=very bad to 10=very good), N: Number, SD: Standard deviation, significant values (p<0.05) are marked by *.
