# Supplementary material for: Self-evaluation of present clinical skills by medical students in the years 3 to 6 – a pilot study in four European countries
Source: GMS J Med Educ. 2018 Aug 15;35(3):Doc36. doi: 10.3205/zma001182 (PMC6120160; doi:10.3205/zma001182)
Supplement: Survey [file JME-35-36-s-001.pdf]

MEDICAL Student Survey

There are 8 questions in this survey

welcome

1

Dear Medical Students,

we are interested in comparing aspects of student experiences from different countries.

We have compiled a brief, on-line survey and hope you are able to spend a few minutes of your valuable time to complete it.

To protect your privacy, answers you provide for the survey are de-identified and cannot be linked to you.

The survey should take less than 5 minutes to complete.

2 In which country are you studying ?

Please choose **only one** of the following:

- ○ Germany
- ○ Switzerland
- ○ United Kingdom
- ○ Austria
- ○ Other [ ]

3 At which university are you studying ?

Please write your answer here:

[ ]

4 What is your age?

Please write your answer here:

[ ]

5 Sex

Please choose **only one** of the following:

- ○ Female
- ○ Male

6 Which year of your medical school are you currently ? *

Please choose **only one** of the following:

- ○ 1
- ○ 2
- ○ 3
- ○ 4
- ○ 5
- ○ 6
- ○ bridging year
- ○ Other

7 How satisfied are you with your medical school? (1 very unsatisfied - 10 very satisfied)

Please choose the appropriate response:

- ○ 1
- ○ 2
- ○ 3
- ○ 4
- ○ 5
- ○ 6
- ○ 7
- ○ 8
- ○ 9
- ○ 10

8 How well does your medical school prepare you your internship?(1 not well at all - 10 excellent)

Please choose the appropriate response:

- ○ 1
- ○ 2
- ○ 3
- ○ 4
- ○ 5
- ○ 6
- ○ 7
- ○ 8
- ○ 9
- ○ 10

9 How would you rate your skills in the following on a scale from 1(very poor) - 10(very good)

Please choose the appropriate response for each item:

	1	2	3	4	5	6	7	8	9	10
patient interview	○	○	○	○	○	○	○	○	○	○
physical examination	s ○	○	○	○	○	○	○	○	○	○
ausculation	○	○	○	○	○	○	○	○	○	○
taking blood	○	○	○	○	○	○	○	○	○	○
laying a canula	○	○	○	○	○	○	○	○	○	○
surgical suture / stiching	○	○	○	○	○	○	○	○	○	○
application of a cast / dressing	○	○	○	○	○	○	○	○	○	○

Thank you for completing this survey.
